# 10-Benzyl-9-(4-eth­oxy­phen­yl)-3,3,6,6-tetra­methyl-3,4,6,7,9,10-hexa­hydro­acridine-1,8(2*H*,5*H*)-dione

**DOI:** 10.1107/S1600536812036094

**Published:** 2012-08-23

**Authors:** V. Sughanya, N. Sureshbabu

**Affiliations:** a Department of Chemistry, Annamalai University, Annamalai Nagar 608 002, Tamil Nadu, India

## Abstract

In the title compound, C_32_H_37_NO_3_, the central dihydro­pyridine ring adopts a nearly planar flattened-boat conformation, whereas both cyclo­hexenone rings adopt half-chair conformations. The mean and maximum deviations from the mean plane of the dihydro­pyridine ring are 0.1252 (9) and 0.188 (1) Å, respectively. The 4-eth­oxy­phenyl and phenyl rings form dihedral angles of 75.20 (4) and 82.14 (5)° with the dihydro­pyridine mean plane, respectively.

## Related literature

For general background, see: Wysocka-Skrzela & Ledochowski (1976 ▶); Nasim & Brychcy (1979 ▶); Thull & Testa (1994 ▶); Reil *et al.* (1994 ▶); Mandi *et al.* (1994 ▶). For related structures, see: Abdelhamid *et al.* (2011 ▶); Khalilov *et al.* (2011 ▶); Tang *et al.* (2008 ▶); Tu *et al.* (2004 ▶). For a related synthesis, see: Li *et al.* (2003 ▶). For ring-puckering parameters, see: Cremer & Pople (1975 ▶). For bond-length data, see: Allen *et al.* (1987 ▶).
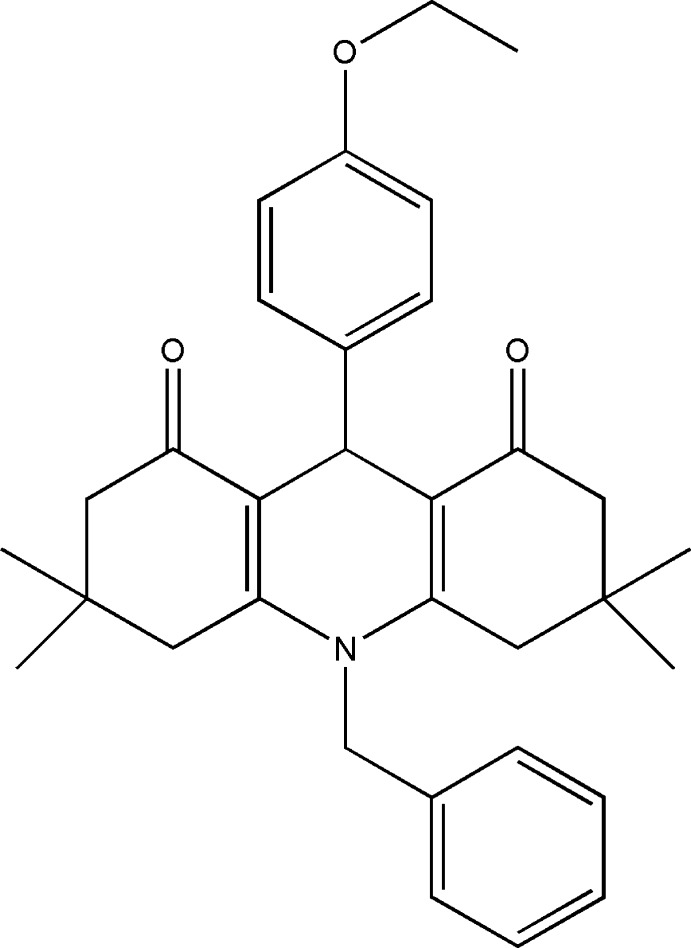


## Experimental

### 

#### Crystal data

C_32_H_37_NO_3_
*M* *_r_* = 483.63
Orthorhombic, 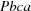

*a* = 16.8172 (7) Å
*b* = 15.7033 (7) Å
*c* = 19.908 (1) Å
*V* = 5257.4 (4) Å^3^
*Z* = 8
Mo *K*α radiation
μ = 0.08 mm^−1^
*T* = 296 K
0.30 × 0.20 × 0.20 mm

#### Data collection

Bruker APEXII CCD diffractometer
Absorption correction: multi-scan (*SADABS*; Bruker, 2004 ▶) *T*_min_ = 0.976, *T*_max_ = 0.986
54483 measured reflections
6422 independent reflections
4439 reflections with *I* > 2σ(*I*)
*R*_int_ = 0.040

#### Refinement

*R*[*F* ^2^ > 2σ(*F* ^2^)] = 0.044
*wR*(*F* ^2^) = 0.128
*S* = 1.03
6422 reflections
326 parameters
H-atom parameters constrained
Δρ_max_ = 0.25 e Å^−3^
Δρ_min_ = −0.16 e Å^−3^

### 

Data collection: *APEX2* (Bruker, 2004 ▶); cell refinement: *APEX2*/*SAINT* (Bruker, 2004 ▶); data reduction: *SAINT*/*XPREP* (Bruker, 2004 ▶); program(s) used to solve structure: *SIR92* (Altomare *et al.*, 1993 ▶); program(s) used to refine structure: *SHELXL97* (Sheldrick, 2008 ▶); molecular graphics: *ORTEP-3* (Farrugia, 1997 ▶) and *Mercury* (Bruno *et al.*, 2002 ▶); software used to prepare material for publication: *SHELXL97*.

## Supplementary Material

Crystal structure: contains datablock(s) global, I. DOI: 10.1107/S1600536812036094/fy2066sup1.cif

Structure factors: contains datablock(s) I. DOI: 10.1107/S1600536812036094/fy2066Isup2.hkl

Additional supplementary materials:  crystallographic information; 3D view; checkCIF report

## Figures and Tables

**Fig. 1.**
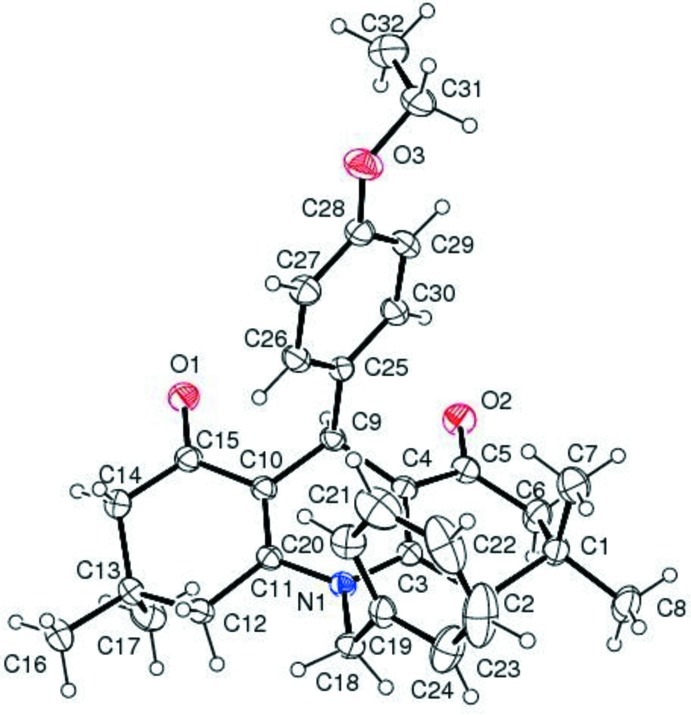
A view of the structure of the title compound showing the atom numbering scheme. Displacement ellipsoids are drawn at the 50% probability level.

**Fig. 2.**
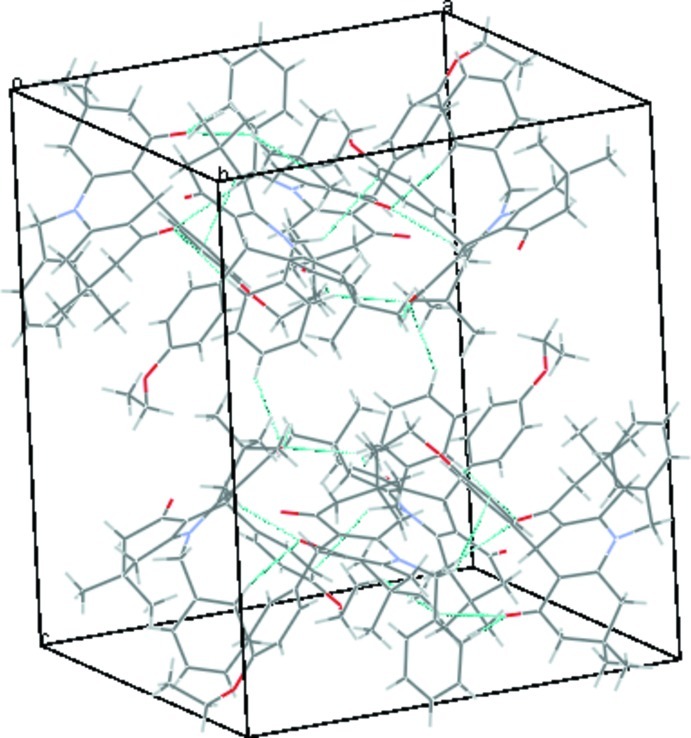
Packing diagram for the title compound.

## supplementary crystallographic information

### Comment

Acridine derivatives with a dihydropyridine unit belong to a special class of compounds, because of their wide range of applications in the pharmaceutical and dye industries. They are also well known as therapeutic agents (Wysocka-Skrzela & Ledochowski, 1976; Nasim & Brychcy,1979; Thull & Testa, 1994; Reil *et al.*, 1994; Mandi *et al.*, 1994).

The central dihydropyridine ring is almost planar with a mean deviation from the mean plane of 0.1252 (9) Å and with a maximum deviation of 0.188 (1) Å for C9. The planar 4-ethoxyphenyl and phenyl rings form dihedral angles of 75.20 (4)° and 82.14 (5)° with the dihydropyridine mean plane. The rings A (C1–C6), B (N1/C3/C4/C9/C10/C11) and C (C10–C15) show total puckering amplitudes Q(T) of 0.506 (2) Å, 0.307 (1) Å and 0.470 (2) Å, respectively. The cyclohexenone rings A and C adopt half chair conformation, whereas the central ring B adopts flattened boat conformation. This can be understood from the Cremer & Pople (1975) puckering parameters: φ = = -7.10 (2)° and θ = 62.2 (2)° (for A); φ = 166.5 (2)°, and θ = 77.4 (2)° (for B) and φ = -163.97 (3)°, θ = 56.9 (2)° (for C), respectively. In the title compound, the bond lengths (Allen *et al.*, 1987) and angles are generally within normal ranges. In the dihydropyridine ring C10=C11 and C4=C3 are double bonds (C10—C11 = 1.3489 (18) Å and C4—C3 = 1.3540 (18) Å), as indicated by the bond distances. The C11—C10—C15 [120.39 (13)°] and C3—C4—C5 [119.51 (13)°] angles are almost the same. In this conformation C1 and C13 may be described as flap atoms being away from the plane of the ring. The observed carbonyl bond lengths (C15—O1 = 1.2232 (17) Å and C5—O2 = 1.2275 (17) Å) are also normal.

### Experimental

The title compound was prepared in two stages. In the first stage, a mixture of 4-ethoxybenzaldehyde (1.2 g, 8 mmol), 5,5-dimethylcyclohexane-1,3-dione (2.24 g, 16 mmol) and 20 ml of ethanol was heated to 70°C for about 10 minutes. The reaction mixture was allowed to cool to room temperature and the resulting solid intermediate, 2,2'-((4-ethoxyphenyl)methylene)bis(3-hydroxy-5,5-dimethylcyclohex-2-enone) was filtered and dried (m.p.: 411 K, yield: 78%). In the second stage about 0.8 g of this intermediate was dissoloved in 25 ml of acetic acid. The solution was refluxed together with benzylamine (0.33 g, 3 mmol) for 8 h with the reaction being monitored by TLC. After completion of the reaction, the reaction mixture was poured into crushed ice and stirred well. The solid that separated was filtered and dried and then recrystallized from ethanol to yield yellow crystals of the title compound (m.p.: 433 K, yield: 82%).

### Refinement

All the hydrogen atoms were fixed in calculated positions and allowed to ride on their parent atom with d(C—H) = 0.96 Å and *U*_iso_(H) = 1.5 *U*_eq_(C) for CH_3_, d(C—H) = 0.97 Å and *U*_iso_(H) = 1.2 *U*_eq_(C) for CH_2_, d(C—H) = 0.98 Å and *U*_iso_(H) = 1.2 *U*_eq_(C) for aliphatic CH and with d(C—H) = 0.93 Å and *U*_iso_(H) = 1.2 *U*_eq_(C) for aromatic CH.

### Crystal data

**Table:**

C_32_H_37_NO_3_	*D*_x_ = 1.222 Mg m^−^^3^
*M**_r_* = 483.63	Mo *K*α radiation, λ = 0.71073 Å
Orthorhombic, *P**b**c**a*	Cell parameters from 6423 reflections
*a* = 16.8172 (7) Å	θ = 2.4–27.3°
*b* = 15.7033 (7) Å	µ = 0.08 mm^−^^1^
*c* = 19.908 (1) Å	*T* = 296 K
*V* = 5257.4 (4) Å^3^	Block, colourless
*Z* = 8	0.30 × 0.20 × 0.20 mm
*F*(000) = 2080	

### Data collection

**Table:**

Bruker APEXII CCD diffractometer	6422 independent reflections
Radiation source: fine-focus sealed tube	4439 reflections with *I* > 2σ(*I*)
Graphite monochromator	*R*_int_ = 0.040
ω and φ scan	θ_max_ = 28.1°, θ_min_ = 2.1°
Absorption correction: multi-scan (*SADABS*; Bruker, 2004)	*h* = −22→22
*T*_min_ = 0.976, *T*_max_ = 0.986	*k* = −20→20
54483 measured reflections	*l* = −26→26

### Refinement

**Table:**

Refinement on *F*^2^	Secondary atom site location: difference Fourier map
Least-squares matrix: full	Hydrogen site location: inferred from neighbouring sites
*R*[*F*^2^ > 2σ(*F*^2^)] = 0.044	H-atom parameters constrained
*wR*(*F*^2^) = 0.128	*w* = 1/[σ^2^(*F*_o_^2^) + (0.0534*P*)^2^ + 1.5474*P*] where *P* = (*F*_o_^2^ + 2*F*_c_^2^)/3
*S* = 1.03	(Δ/σ)_max_ < 0.001
6422 reflections	Δρ_max_ = 0.25 e Å^−^^3^
326 parameters	Δρ_min_ = −0.16 e Å^−^^3^
0 restraints	Extinction correction: *SHELXL97*, Fc^*^=kFc[1+0.001xFc^2^λ^3^/sin(2θ)]^-1/4^
Primary atom site location: structure-invariant direct methods	Extinction coefficient: 0.0017 (3)

### Special details

**Table:**

Geometry. All e.s.d.'s (except the e.s.d. in the dihedral angle between two l.s. planes) are estimated using the full covariance matrix. The cell e.s.d.'s are taken into account individually in the estimation of e.s.d.'s in distances, angles and torsion angles; correlations between e.s.d.'s in cell parameters are only used when they are defined by crystal symmetry. An approximate (isotropic) treatment of cell e.s.d.'s is used for estimating e.s.d.'s involving l.s. planes.
Refinement. Refinement of *F*^2^ against ALL reflections. The weighted *R*-factor *wR* and goodness of fit *S* are based on *F*^2^, conventional *R*-factors *R* are based on *F*, with *F* set to zero for negative *F*^2^. The threshold expression of *F*^2^ > σ(*F*^2^) is used only for calculating *R*-factors(gt) *etc*. and is not relevant to the choice of reflections for refinement. *R*-factors based on *F*^2^ are statistically about twice as large as those based on *F*, and *R*- factors based on ALL data will be even larger.

### Fractional atomic coordinates and isotropic or equivalent isotropic displacement parameters (Å^2^)

**Table:**

	*x*	*y*	*z*	*U*_iso_*/*U*_eq_	
C1	0.43933 (9)	0.70202 (10)	0.33199 (8)	0.0427 (4)	
C2	0.44922 (8)	0.61875 (9)	0.29361 (8)	0.0386 (3)	
H2A	0.4920	0.6251	0.2613	0.046*	
H2B	0.4644	0.5743	0.3249	0.046*	
C3	0.37519 (8)	0.59175 (8)	0.25718 (7)	0.0308 (3)	
C4	0.31496 (8)	0.64638 (8)	0.24372 (7)	0.0323 (3)	
C5	0.32733 (9)	0.73706 (9)	0.25208 (7)	0.0386 (3)	
C6	0.40380 (10)	0.76626 (10)	0.28343 (9)	0.0512 (4)	
H6A	0.3943	0.8192	0.3072	0.061*	
H6B	0.4421	0.7777	0.2481	0.061*	
C7	0.38514 (12)	0.68989 (15)	0.39259 (9)	0.0668 (5)	
H7A	0.4084	0.6491	0.4227	0.100*	
H7B	0.3342	0.6696	0.3779	0.100*	
H7C	0.3787	0.7433	0.4154	0.100*	
C8	0.52092 (11)	0.73258 (12)	0.35585 (11)	0.0629 (5)	
H8A	0.5430	0.6916	0.3863	0.094*	
H8B	0.5154	0.7864	0.3783	0.094*	
H8C	0.5556	0.7390	0.3179	0.094*	
C9	0.23605 (8)	0.61457 (9)	0.21724 (7)	0.0325 (3)	
H9	0.2149	0.6583	0.1870	0.039*	
C10	0.25047 (8)	0.53568 (9)	0.17623 (7)	0.0329 (3)	
C11	0.31540 (8)	0.48656 (8)	0.18523 (6)	0.0307 (3)	
C12	0.33256 (8)	0.41003 (9)	0.14247 (7)	0.0362 (3)	
H12A	0.3216	0.3592	0.1685	0.043*	
H12B	0.3887	0.4097	0.1314	0.043*	
C13	0.28452 (9)	0.40604 (10)	0.07704 (7)	0.0418 (4)	
C14	0.19796 (9)	0.42652 (11)	0.09332 (8)	0.0462 (4)	
H14A	0.1676	0.4279	0.0519	0.055*	
H14B	0.1763	0.3816	0.1213	0.055*	
C15	0.18853 (8)	0.51014 (10)	0.12889 (7)	0.0382 (3)	
C16	0.29115 (11)	0.31582 (12)	0.04862 (10)	0.0615 (5)	
H16A	0.2715	0.2758	0.0810	0.092*	
H16B	0.3458	0.3034	0.0388	0.092*	
H16C	0.2603	0.3117	0.0082	0.092*	
C17	0.31637 (12)	0.46944 (13)	0.02556 (8)	0.0594 (5)	
H17A	0.3709	0.4563	0.0156	0.089*	
H17B	0.3130	0.5261	0.0435	0.089*	
H17C	0.2853	0.4659	−0.0148	0.089*	
C18	0.42660 (9)	0.44408 (9)	0.26170 (8)	0.0385 (3)	
H18A	0.4806	0.4657	0.2601	0.046*	
H18B	0.4244	0.3932	0.2341	0.046*	
C19	0.40643 (9)	0.42094 (9)	0.33302 (8)	0.0390 (3)	
C20	0.32955 (11)	0.39930 (11)	0.35078 (9)	0.0523 (4)	
H20	0.2897	0.3994	0.3184	0.063*	
C21	0.31140 (15)	0.37766 (14)	0.41603 (11)	0.0765 (6)	
H21	0.2596	0.3626	0.4271	0.092*	
C22	0.3683 (2)	0.37810 (14)	0.46431 (11)	0.0875 (8)	
H22	0.3555	0.3637	0.5083	0.105*	
C23	0.4439 (2)	0.39963 (15)	0.44815 (12)	0.0943 (9)	
H23	0.4827	0.4008	0.4814	0.113*	
C24	0.46399 (13)	0.42012 (12)	0.38220 (11)	0.0687 (6)	
H24	0.5164	0.4333	0.3714	0.082*	
C25	0.17591 (8)	0.60221 (9)	0.27392 (7)	0.0339 (3)	
C26	0.14725 (9)	0.52322 (10)	0.29309 (7)	0.0396 (3)	
H26	0.1657	0.4746	0.2715	0.048*	
C27	0.09150 (9)	0.51533 (10)	0.34395 (8)	0.0436 (4)	
H27	0.0730	0.4616	0.3560	0.052*	
C28	0.06319 (9)	0.58640 (11)	0.37696 (8)	0.0428 (4)	
C29	0.09195 (9)	0.66542 (11)	0.35946 (9)	0.0500 (4)	
H29	0.0743	0.7138	0.3819	0.060*	
C30	0.14731 (9)	0.67259 (10)	0.30830 (8)	0.0453 (4)	
H30	0.1659	0.7264	0.2966	0.054*	
C31	−0.02477 (11)	0.64092 (14)	0.46208 (9)	0.0619 (5)	
H31A	0.0179	0.6797	0.4739	0.074*	
H31B	−0.0482	0.6201	0.5035	0.074*	
C32	−0.08674 (12)	0.68881 (16)	0.42322 (11)	0.0756 (6)	
H32A	−0.1065	0.7351	0.4500	0.113*	
H32B	−0.1297	0.6511	0.4121	0.113*	
H32C	−0.0637	0.7109	0.3827	0.113*	
N1	0.37233 (7)	0.50849 (7)	0.23366 (6)	0.0327 (3)	
O1	0.12873 (7)	0.55348 (8)	0.12149 (6)	0.0547 (3)	
O2	0.27853 (7)	0.78993 (7)	0.23301 (6)	0.0527 (3)	
O3	0.00768 (7)	0.57079 (9)	0.42588 (6)	0.0605 (3)	

### Atomic displacement parameters (Å^2^)

**Table:**

	*U*^11^	*U*^22^	*U*^33^	*U*^12^	*U*^13^	*U*^23^
C1	0.0415 (8)	0.0388 (8)	0.0478 (9)	−0.0022 (6)	−0.0073 (7)	−0.0038 (7)
C2	0.0337 (7)	0.0346 (8)	0.0475 (8)	−0.0009 (6)	−0.0024 (6)	0.0010 (6)
C3	0.0325 (7)	0.0302 (7)	0.0298 (7)	−0.0012 (5)	0.0028 (5)	0.0021 (5)
C4	0.0338 (7)	0.0312 (7)	0.0319 (7)	0.0020 (5)	0.0015 (5)	0.0009 (5)
C5	0.0428 (8)	0.0332 (7)	0.0397 (8)	0.0031 (6)	0.0025 (6)	0.0031 (6)
C6	0.0535 (9)	0.0308 (8)	0.0693 (11)	−0.0031 (7)	−0.0105 (8)	−0.0004 (7)
C7	0.0682 (12)	0.0884 (15)	0.0437 (10)	−0.0004 (11)	−0.0013 (9)	−0.0126 (10)
C8	0.0554 (10)	0.0464 (10)	0.0868 (14)	−0.0040 (8)	−0.0236 (10)	−0.0120 (9)
C9	0.0330 (7)	0.0325 (7)	0.0321 (7)	0.0061 (5)	−0.0010 (5)	−0.0012 (5)
C10	0.0329 (6)	0.0353 (7)	0.0306 (7)	0.0019 (6)	0.0018 (5)	−0.0015 (5)
C11	0.0329 (6)	0.0308 (7)	0.0284 (6)	−0.0001 (5)	0.0044 (5)	0.0009 (5)
C12	0.0344 (7)	0.0354 (8)	0.0389 (7)	0.0024 (6)	0.0055 (6)	−0.0046 (6)
C13	0.0422 (8)	0.0451 (9)	0.0383 (8)	0.0018 (7)	0.0019 (6)	−0.0116 (7)
C14	0.0397 (8)	0.0525 (10)	0.0465 (9)	−0.0004 (7)	−0.0046 (7)	−0.0121 (7)
C15	0.0353 (7)	0.0461 (9)	0.0332 (7)	0.0029 (6)	0.0009 (6)	−0.0022 (6)
C16	0.0597 (11)	0.0598 (11)	0.0650 (12)	0.0051 (9)	−0.0049 (9)	−0.0286 (9)
C17	0.0684 (12)	0.0712 (12)	0.0386 (9)	0.0050 (10)	0.0105 (8)	0.0003 (8)
C18	0.0363 (7)	0.0323 (7)	0.0470 (8)	0.0082 (6)	−0.0030 (6)	−0.0009 (6)
C19	0.0482 (8)	0.0245 (7)	0.0443 (8)	0.0033 (6)	−0.0097 (7)	−0.0009 (6)
C20	0.0543 (10)	0.0492 (10)	0.0534 (10)	0.0019 (8)	−0.0001 (8)	0.0036 (8)
C21	0.0986 (17)	0.0636 (13)	0.0672 (14)	0.0018 (12)	0.0270 (13)	0.0092 (10)
C22	0.162 (3)	0.0540 (13)	0.0459 (11)	−0.0053 (15)	0.0005 (15)	0.0041 (9)
C23	0.152 (3)	0.0677 (15)	0.0630 (14)	−0.0223 (16)	−0.0547 (16)	0.0181 (11)
C24	0.0751 (13)	0.0590 (12)	0.0719 (13)	−0.0124 (10)	−0.0339 (11)	0.0174 (10)
C25	0.0295 (6)	0.0380 (8)	0.0343 (7)	0.0033 (6)	−0.0017 (5)	−0.0050 (6)
C26	0.0416 (8)	0.0384 (8)	0.0390 (8)	0.0066 (6)	0.0021 (6)	−0.0044 (6)
C27	0.0453 (8)	0.0424 (9)	0.0430 (8)	0.0002 (7)	0.0037 (7)	0.0017 (7)
C28	0.0376 (7)	0.0561 (10)	0.0347 (8)	0.0011 (7)	0.0031 (6)	−0.0045 (7)
C29	0.0464 (9)	0.0493 (10)	0.0541 (10)	0.0008 (7)	0.0115 (7)	−0.0199 (8)
C30	0.0431 (8)	0.0383 (8)	0.0545 (9)	−0.0022 (7)	0.0073 (7)	−0.0115 (7)
C31	0.0512 (10)	0.0908 (15)	0.0437 (9)	0.0045 (10)	0.0093 (8)	−0.0171 (9)
C32	0.0558 (11)	0.0931 (16)	0.0777 (14)	0.0088 (11)	0.0036 (10)	−0.0102 (12)
N1	0.0338 (6)	0.0297 (6)	0.0347 (6)	0.0041 (5)	−0.0014 (5)	−0.0003 (5)
O1	0.0443 (6)	0.0648 (8)	0.0551 (7)	0.0161 (6)	−0.0139 (5)	−0.0119 (6)
O2	0.0547 (7)	0.0340 (6)	0.0693 (8)	0.0090 (5)	−0.0071 (6)	0.0060 (5)
O3	0.0585 (7)	0.0716 (9)	0.0515 (7)	0.0025 (6)	0.0200 (6)	−0.0040 (6)

### Geometric parameters (Å, º)

**Table:**

C1—C6	1.520 (2)	C16—H16B	0.9600
C1—C2	1.523 (2)	C16—H16C	0.9600
C1—C7	1.524 (2)	C17—H17A	0.9600
C1—C8	1.529 (2)	C17—H17B	0.9600
C2—C3	1.5020 (19)	C17—H17C	0.9600
C2—H2A	0.9700	C18—N1	1.4723 (17)
C2—H2B	0.9700	C18—C19	1.504 (2)
C3—C4	1.3540 (18)	C18—H18A	0.9700
C3—N1	1.3896 (17)	C18—H18B	0.9700
C4—C5	1.449 (2)	C19—C24	1.377 (2)
C4—C9	1.5127 (19)	C19—C20	1.383 (2)
C5—O2	1.2275 (17)	C20—C21	1.377 (3)
C5—C6	1.501 (2)	C20—H20	0.9300
C6—H6A	0.9700	C21—C22	1.357 (3)
C6—H6B	0.9700	C21—H21	0.9300
C7—H7A	0.9600	C22—C23	1.353 (4)
C7—H7B	0.9600	C22—H22	0.9300
C7—H7C	0.9600	C23—C24	1.394 (3)
C8—H8A	0.9600	C23—H23	0.9300
C8—H8B	0.9600	C24—H24	0.9300
C8—H8C	0.9600	C25—C26	1.384 (2)
C9—C10	1.5033 (19)	C25—C30	1.386 (2)
C9—C25	1.5277 (19)	C26—C27	1.385 (2)
C9—H9	0.9800	C26—H26	0.9300
C10—C11	1.3489 (18)	C27—C28	1.380 (2)
C10—C15	1.4609 (19)	C27—H27	0.9300
C11—N1	1.4017 (17)	C28—O3	1.3711 (19)
C11—C12	1.5007 (18)	C28—C29	1.377 (2)
C12—C13	1.534 (2)	C29—C30	1.384 (2)
C12—H12A	0.9700	C29—H29	0.9300
C12—H12B	0.9700	C30—H30	0.9300
C13—C14	1.526 (2)	C31—O3	1.425 (2)
C13—C17	1.526 (2)	C31—C32	1.500 (3)
C13—C16	1.530 (2)	C31—H31A	0.9700
C14—C15	1.500 (2)	C31—H31B	0.9700
C14—H14A	0.9700	C32—H32A	0.9600
C14—H14B	0.9700	C32—H32B	0.9600
C15—O1	1.2232 (17)	C32—H32C	0.9600
C16—H16A	0.9600		
			
C6—C1—C2	107.08 (13)	C10—C15—C14	118.00 (12)
C6—C1—C7	110.58 (15)	C13—C16—H16A	109.5
C2—C1—C7	110.80 (14)	C13—C16—H16B	109.5
C6—C1—C8	110.00 (14)	H16A—C16—H16B	109.5
C2—C1—C8	109.09 (13)	C13—C16—H16C	109.5
C7—C1—C8	109.26 (15)	H16A—C16—H16C	109.5
C3—C2—C1	113.20 (12)	H16B—C16—H16C	109.5
C3—C2—H2A	108.9	C13—C17—H17A	109.5
C1—C2—H2A	108.9	C13—C17—H17B	109.5
C3—C2—H2B	108.9	H17A—C17—H17B	109.5
C1—C2—H2B	108.9	C13—C17—H17C	109.5
H2A—C2—H2B	107.8	H17A—C17—H17C	109.5
C4—C3—N1	120.23 (12)	H17B—C17—H17C	109.5
C4—C3—C2	122.47 (12)	N1—C18—C19	112.58 (12)
N1—C3—C2	117.19 (11)	N1—C18—H18A	109.1
C3—C4—C5	119.51 (13)	C19—C18—H18A	109.1
C3—C4—C9	121.07 (12)	N1—C18—H18B	109.1
C5—C4—C9	119.37 (12)	C19—C18—H18B	109.1
O2—C5—C4	122.23 (14)	H18A—C18—H18B	107.8
O2—C5—C6	119.65 (13)	C24—C19—C20	118.25 (17)
C4—C5—C6	118.10 (13)	C24—C19—C18	120.99 (16)
C5—C6—C1	113.49 (13)	C20—C19—C18	120.76 (14)
C5—C6—H6A	108.9	C21—C20—C19	120.61 (19)
C1—C6—H6A	108.9	C21—C20—H20	119.7
C5—C6—H6B	108.9	C19—C20—H20	119.7
C1—C6—H6B	108.9	C22—C21—C20	120.7 (2)
H6A—C6—H6B	107.7	C22—C21—H21	119.7
C1—C7—H7A	109.5	C20—C21—H21	119.7
C1—C7—H7B	109.5	C23—C22—C21	119.7 (2)
H7A—C7—H7B	109.5	C23—C22—H22	120.1
C1—C7—H7C	109.5	C21—C22—H22	120.1
H7A—C7—H7C	109.5	C22—C23—C24	120.6 (2)
H7B—C7—H7C	109.5	C22—C23—H23	119.7
C1—C8—H8A	109.5	C24—C23—H23	119.7
C1—C8—H8B	109.5	C19—C24—C23	120.1 (2)
H8A—C8—H8B	109.5	C19—C24—H24	120.0
C1—C8—H8C	109.5	C23—C24—H24	120.0
H8A—C8—H8C	109.5	C26—C25—C30	117.22 (13)
H8B—C8—H8C	109.5	C26—C25—C9	123.23 (12)
C10—C9—C4	108.65 (11)	C30—C25—C9	119.55 (13)
C10—C9—C25	113.78 (12)	C25—C26—C27	121.14 (14)
C4—C9—C25	111.43 (11)	C25—C26—H26	119.4
C10—C9—H9	107.6	C27—C26—H26	119.4
C4—C9—H9	107.6	C28—C27—C26	120.61 (15)
C25—C9—H9	107.6	C28—C27—H27	119.7
C11—C10—C15	120.39 (13)	C26—C27—H27	119.7
C11—C10—C9	121.99 (12)	O3—C28—C29	125.46 (14)
C15—C10—C9	117.48 (12)	O3—C28—C27	115.37 (15)
C10—C11—N1	120.24 (12)	C29—C28—C27	119.16 (14)
C10—C11—C12	122.57 (12)	C28—C29—C30	119.72 (14)
N1—C11—C12	117.11 (11)	C28—C29—H29	120.1
C11—C12—C13	114.40 (12)	C30—C29—H29	120.1
C11—C12—H12A	108.7	C29—C30—C25	122.12 (15)
C13—C12—H12A	108.7	C29—C30—H30	118.9
C11—C12—H12B	108.7	C25—C30—H30	118.9
C13—C12—H12B	108.7	O3—C31—C32	113.12 (16)
H12A—C12—H12B	107.6	O3—C31—H31A	109.0
C14—C13—C17	109.87 (14)	C32—C31—H31A	109.0
C14—C13—C16	110.08 (13)	O3—C31—H31B	109.0
C17—C13—C16	109.29 (14)	C32—C31—H31B	109.0
C14—C13—C12	108.27 (12)	H31A—C31—H31B	107.8
C17—C13—C12	111.03 (13)	C31—C32—H32A	109.5
C16—C13—C12	108.28 (13)	C31—C32—H32B	109.5
C15—C14—C13	112.68 (13)	H32A—C32—H32B	109.5
C15—C14—H14A	109.1	C31—C32—H32C	109.5
C13—C14—H14A	109.1	H32A—C32—H32C	109.5
C15—C14—H14B	109.1	H32B—C32—H32C	109.5
C13—C14—H14B	109.1	C3—N1—C11	119.12 (11)
H14A—C14—H14B	107.8	C3—N1—C18	119.81 (11)
O1—C15—C10	120.74 (13)	C11—N1—C18	121.02 (11)
O1—C15—C14	121.13 (13)	C28—O3—C31	118.81 (15)
			
C6—C1—C2—C3	−50.29 (17)	C9—C10—C15—C14	−173.31 (13)
C7—C1—C2—C3	70.38 (17)	C13—C14—C15—O1	148.98 (15)
C8—C1—C2—C3	−169.30 (14)	C13—C14—C15—C10	−35.1 (2)
C1—C2—C3—C4	18.00 (19)	N1—C18—C19—C24	−130.50 (16)
C1—C2—C3—N1	−165.83 (12)	N1—C18—C19—C20	49.97 (19)
N1—C3—C4—C5	−163.38 (12)	C24—C19—C20—C21	0.1 (3)
C2—C3—C4—C5	12.7 (2)	C18—C19—C20—C21	179.61 (16)
N1—C3—C4—C9	13.97 (19)	C19—C20—C21—C22	0.8 (3)
C2—C3—C4—C9	−169.97 (12)	C20—C21—C22—C23	−0.3 (4)
C3—C4—C5—O2	170.96 (14)	C21—C22—C23—C24	−1.0 (4)
C9—C4—C5—O2	−6.4 (2)	C20—C19—C24—C23	−1.3 (3)
C3—C4—C5—C6	−7.3 (2)	C18—C19—C24—C23	179.12 (18)
C9—C4—C5—C6	175.34 (13)	C22—C23—C24—C19	1.8 (4)
O2—C5—C6—C1	153.19 (15)	C10—C9—C25—C26	10.19 (19)
C4—C5—C6—C1	−28.5 (2)	C4—C9—C25—C26	−113.05 (15)
C2—C1—C6—C5	55.69 (18)	C10—C9—C25—C30	−169.34 (13)
C7—C1—C6—C5	−65.12 (19)	C4—C9—C25—C30	67.41 (17)
C8—C1—C6—C5	174.12 (15)	C30—C25—C26—C27	0.8 (2)
C3—C4—C9—C10	−30.37 (17)	C9—C25—C26—C27	−178.69 (13)
C5—C4—C9—C10	146.99 (13)	C25—C26—C27—C28	−0.1 (2)
C3—C4—C9—C25	95.76 (15)	C26—C27—C28—O3	179.40 (14)
C5—C4—C9—C25	−86.89 (15)	C26—C27—C28—C29	−1.0 (2)
C4—C9—C10—C11	23.49 (18)	O3—C28—C29—C30	−179.07 (15)
C25—C9—C10—C11	−101.26 (15)	C27—C28—C29—C30	1.4 (2)
C4—C9—C10—C15	−160.71 (12)	C28—C29—C30—C25	−0.7 (3)
C25—C9—C10—C15	74.54 (15)	C26—C25—C30—C29	−0.5 (2)
C15—C10—C11—N1	−176.05 (12)	C9—C25—C30—C29	179.10 (14)
C9—C10—C11—N1	−0.4 (2)	C4—C3—N1—C11	12.70 (18)
C15—C10—C11—C12	7.3 (2)	C2—C3—N1—C11	−163.56 (12)
C9—C10—C11—C12	−176.98 (12)	C4—C3—N1—C18	−164.86 (12)
C10—C11—C12—C13	15.91 (19)	C2—C3—N1—C18	18.88 (18)
N1—C11—C12—C13	−160.80 (12)	C10—C11—N1—C3	−19.72 (18)
C11—C12—C13—C14	−45.85 (17)	C12—C11—N1—C3	157.07 (12)
C11—C12—C13—C17	74.84 (16)	C10—C11—N1—C18	157.81 (13)
C11—C12—C13—C16	−165.18 (13)	C12—C11—N1—C18	−25.40 (18)
C17—C13—C14—C15	−66.36 (17)	C19—C18—N1—C3	70.01 (16)
C16—C13—C14—C15	173.24 (14)	C19—C18—N1—C11	−107.50 (14)
C12—C13—C14—C15	55.05 (18)	C29—C28—O3—C31	0.7 (2)
C11—C10—C15—O1	178.49 (14)	C27—C28—O3—C31	−179.71 (14)
C9—C10—C15—O1	2.6 (2)	C32—C31—O3—C28	77.7 (2)
C11—C10—C15—C14	2.6 (2)		
